# Supplementary material for: Microdiversity of the vaginal microbiome is associated with preterm birth
Source: Nat Commun. 2023 Aug 17;14:4997. doi: 10.1038/s41467-023-40719-7 (PMC10435516; doi:10.1038/s41467-023-40719-7)
Supplement: Supplementary file 1 — Supplementary Information [file 41467_2023_40719_MOESM1_ESM.pdf]

## Supplementary figures

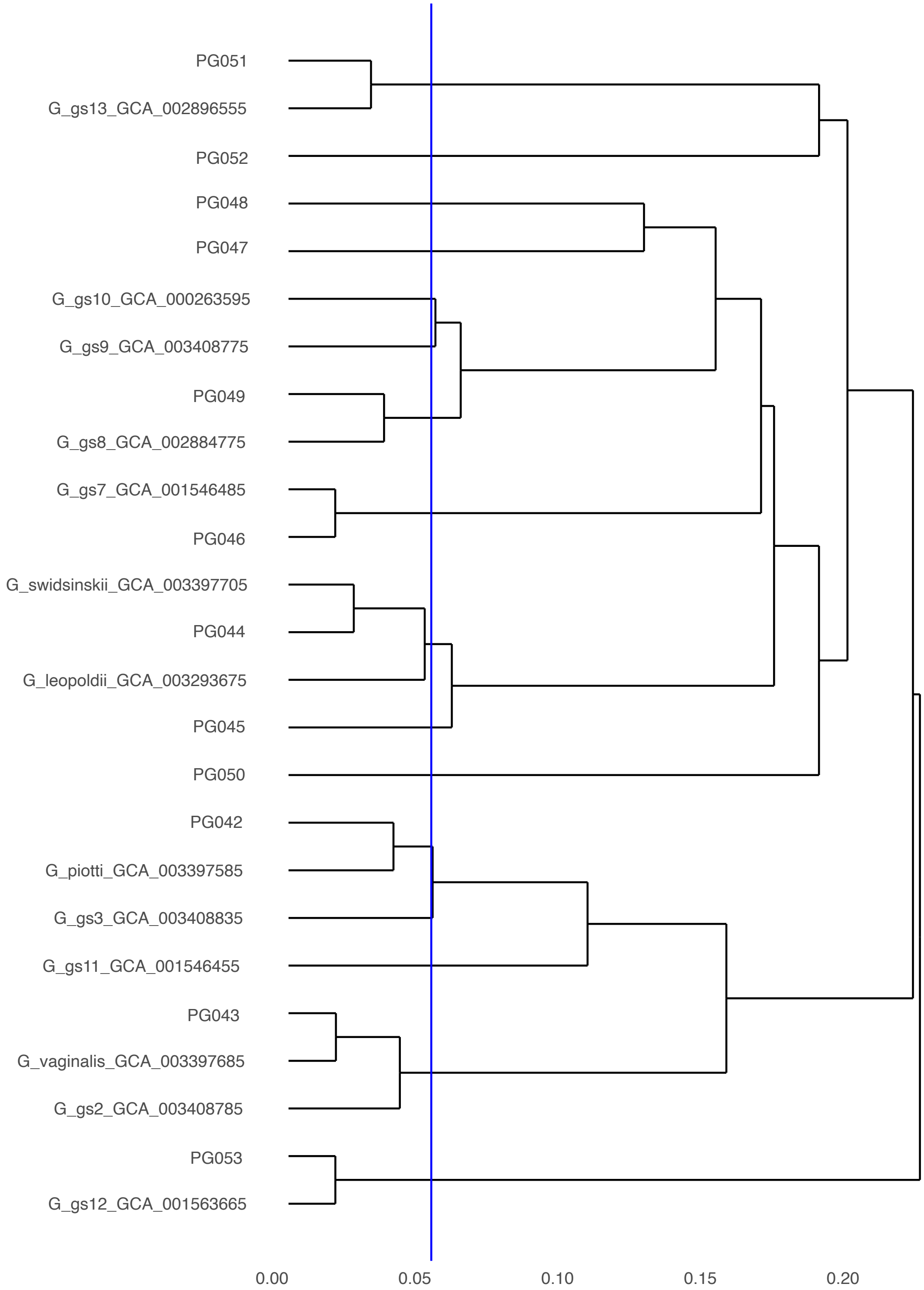


**Supplementary Fig. 1 | Dendrogram of representative MAGs annotated as *G. vaginalis* (PG42-53) and reference genomes of 13 *Gardnerella* species defined in ref.** [25] **based on ANI.** Blue line shows an ANI value of 0.95, which is a cutoff for prokaryotes species.

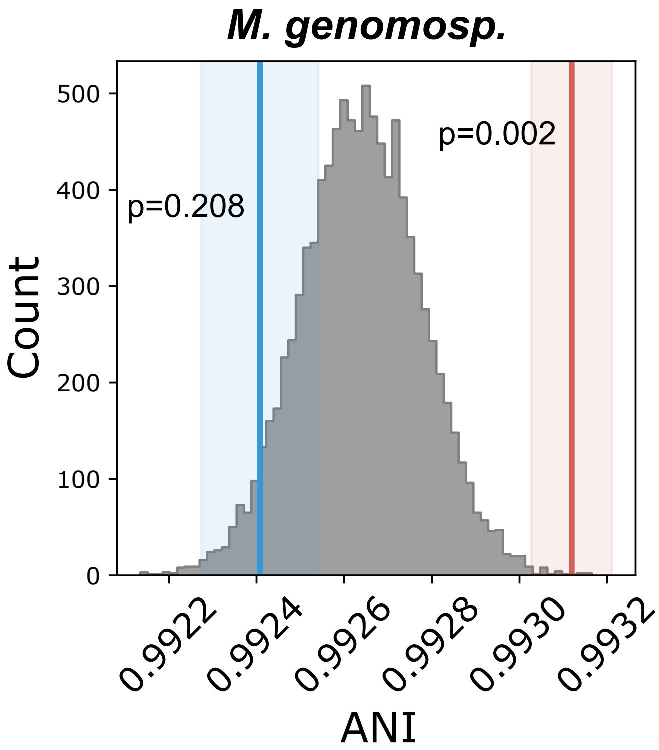


**Supplementary Fig. 2 | The distribution of average inter-host nucleotide identity (ANI) of MAGs classified as *M. genomosp*.** The gray histogram illustrates the null distribution. The red and blue line and shaded area indicate the average value and standard deviation of ANI observed in sPTB and in TB, respectively, calculated from 10,000 repetitions. *p*, one-sided significance vs. null.

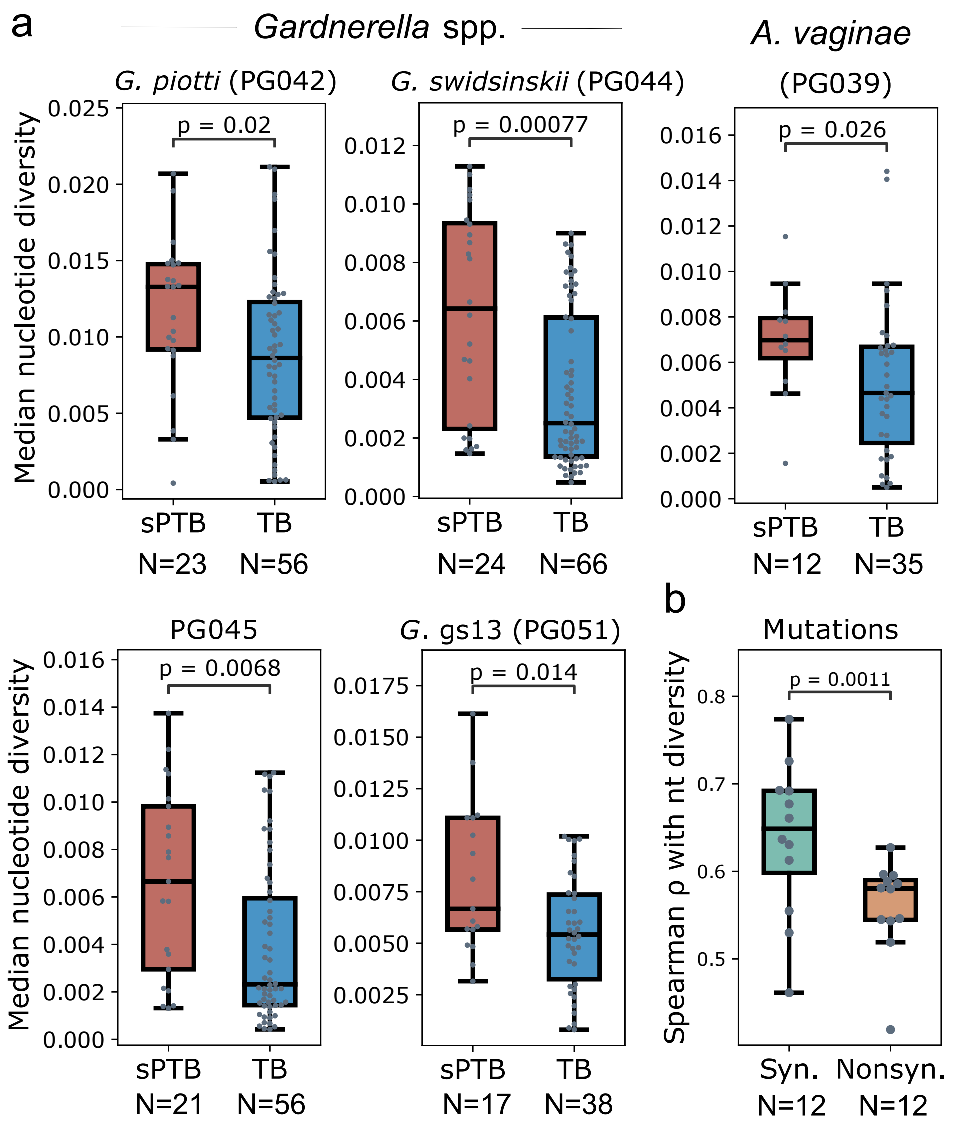


**Supplementary Fig. 3 | Nucleotide diversity of vaginal microbial populations. a.** Median genome-wide nucleotide diversity along pregnancies of four *Gardnerella* spp. phylogroups and a phylogroup classified as *A. vaginae*, compared between sPTB and TB. *p*, two-sided Mann-Whitney U. **b.** Spearman correlation coefficient between nucleotide diversity and number of synonymous mutations and nonsynonymous mutations of genes across 12 *Gardnerella* spp. phylogroups. Median spearman correlation coefficient of genes of each phylogroup based on median gene nucleotide diversity along pregnancy, compared between synonymous (Syn.) mutations and nonsynonymous (Nonsyn.) mutations. *p*, two-sided Student T test. Box, IQR; line, median; whiskers, 1.5*IQR.

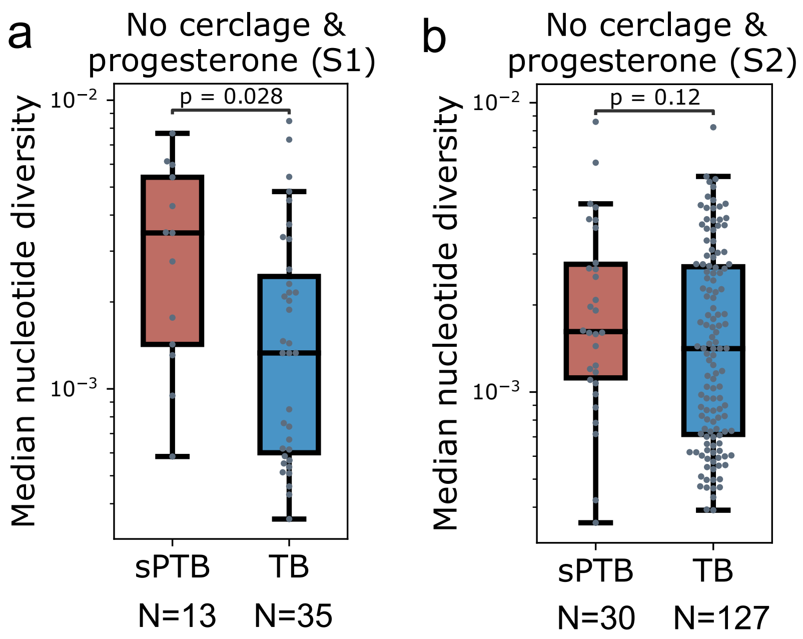


**Supplementary Fig. 4 | The association between microdiversity and sPTB is not biased by medical interventions for risk of preterm birth, including cerclage and progesterone. a, b,** A comparison of median genome-wide nucleotide diversity of *Gardnerella* spp. between sPTB and TB, , for women who did not receive cerclage nor progesterone during pregnancy, displayed for pregnancy S1 (a) and S2 (b). Box, IQR; line, median; whiskers, 1.5*IQR; *p*, two-sided Mann-Whitney.

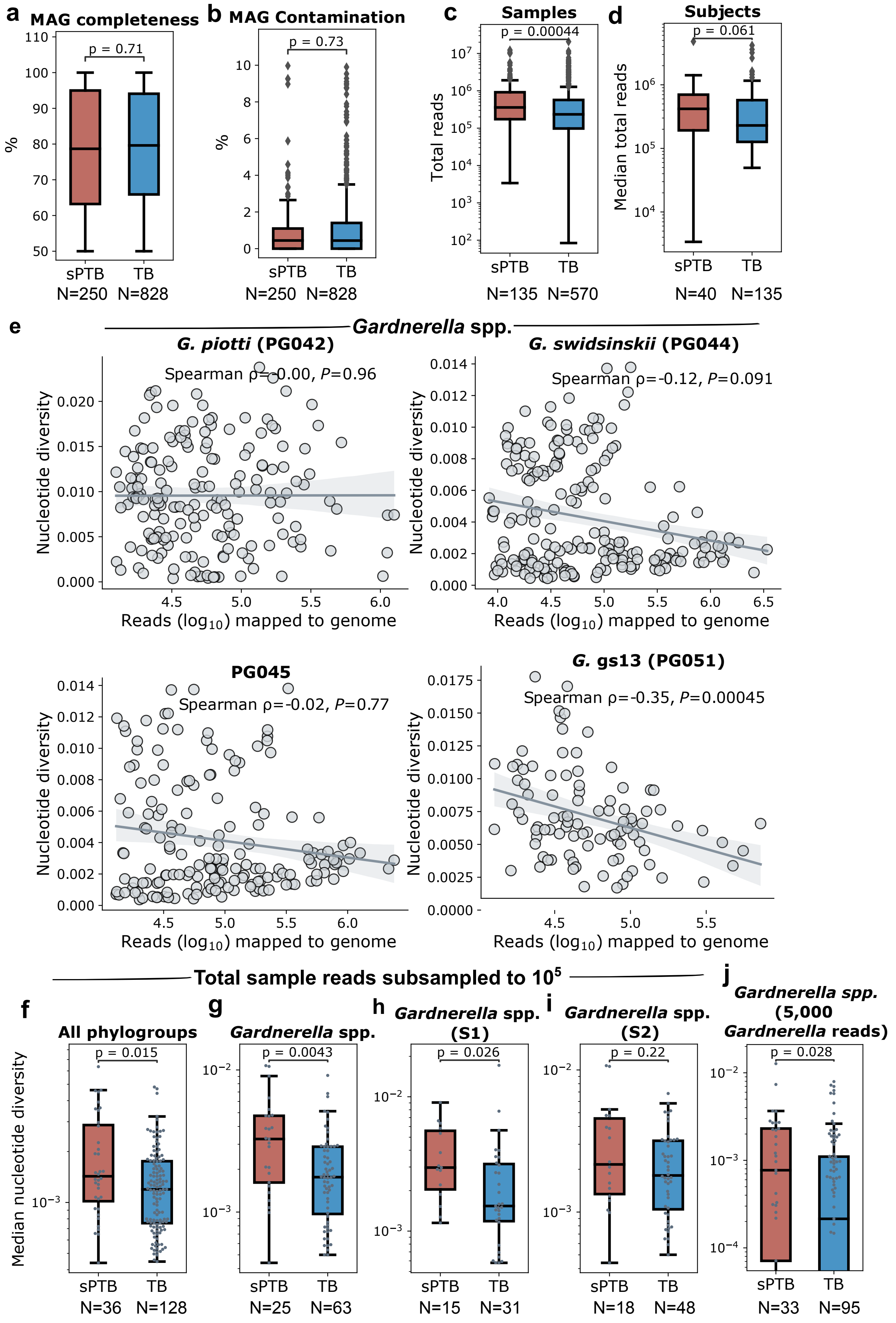

a MAG completeness
p = 0.71
%
sPTB N=250
TB N=828
b MAG Contamination
p = 0.73
%
sPTB N=250
TB N=828
c Samples
p = 0.00044
Total reads
sPTB N=135
TB N=570
d Subjects
p = 0.061
Median total reads
sPTB N=40
TB N=135
e Gardnerella spp.
G. piotti (PG042)
Spearman ρ=-0.00, P=0.96
G. swidsinskii (PG044)
Spearman ρ=-0.12, P=0.091
PG045
Spearman ρ=-0.02, P=0.77
G. gs13 (PG051)
Spearman ρ=-0.35, P=0.00045
Nucleotide diversity
Reads (log10) mapped to genome
Total sample reads subsampled to 10^5
f All phylogroups
p = 0.015
Median nucleotide diversity
sPTB N=36
TB N=128
g Gardnerella spp.
p = 0.0043
sPTB N=25
TB N=63
h Gardnerella spp. (S1)
p = 0.026
sPTB N=15
TB N=31
i Gardnerella spp. (S2)
p = 0.22
sPTB N=18
TB N=48
j Gardnerella spp. (5,000 Gardnerella reads)
p = 0.028
sPTB N=33
TB N=95

**Supplementary Fig. 5 | The association between microdiversity and sPTB is not biased by sequencing depth.** **a,b,** Completeness (a) and contamination (b) of MAGs, compared between sPTB and TB. **c,d,** Total read counts of samples (c) and median read count (d) along each pregnancy compared between sPTB and TB. **e,** Spearman correlation between genome-wide nucleotide diversity and reads mapped to each of the four *Gardnerella* spp. phylogroups that show difference in nucleotide diversity between sPTB and TB in **Fig. S2a**. The line and the shaded area depict the best-fit trendline and the 95% confidence interval (mean ± 1.96 s.e.m.) of the linear regression. **f,g,** Median genome-wide nucleotide diversity along pregnancy of all phylogroups (f) and *Gardnerella* spp. (g), compared between sPTB and TB based on $10^5$ reads sampled from each sample. **h,i,** Median genome-wide nucleotide diversity of *Gardnerella* spp. along the first (S1, h) and second (S2, i) halves of pregnancy, compared between sPTB and TB based on $10^5$ reads sampled from each sample. **j.** Median genome-wide nucleotide diversity along pregnancy of *Gardnerella* spp., compared between sPTB and TB based on 5,000 reads mapped to *Gardnerella* spp. sampled from each sample. Box, IQR; line, median; whiskers, 1.5*IQR; *p*, two-sided Mann-Whitney.

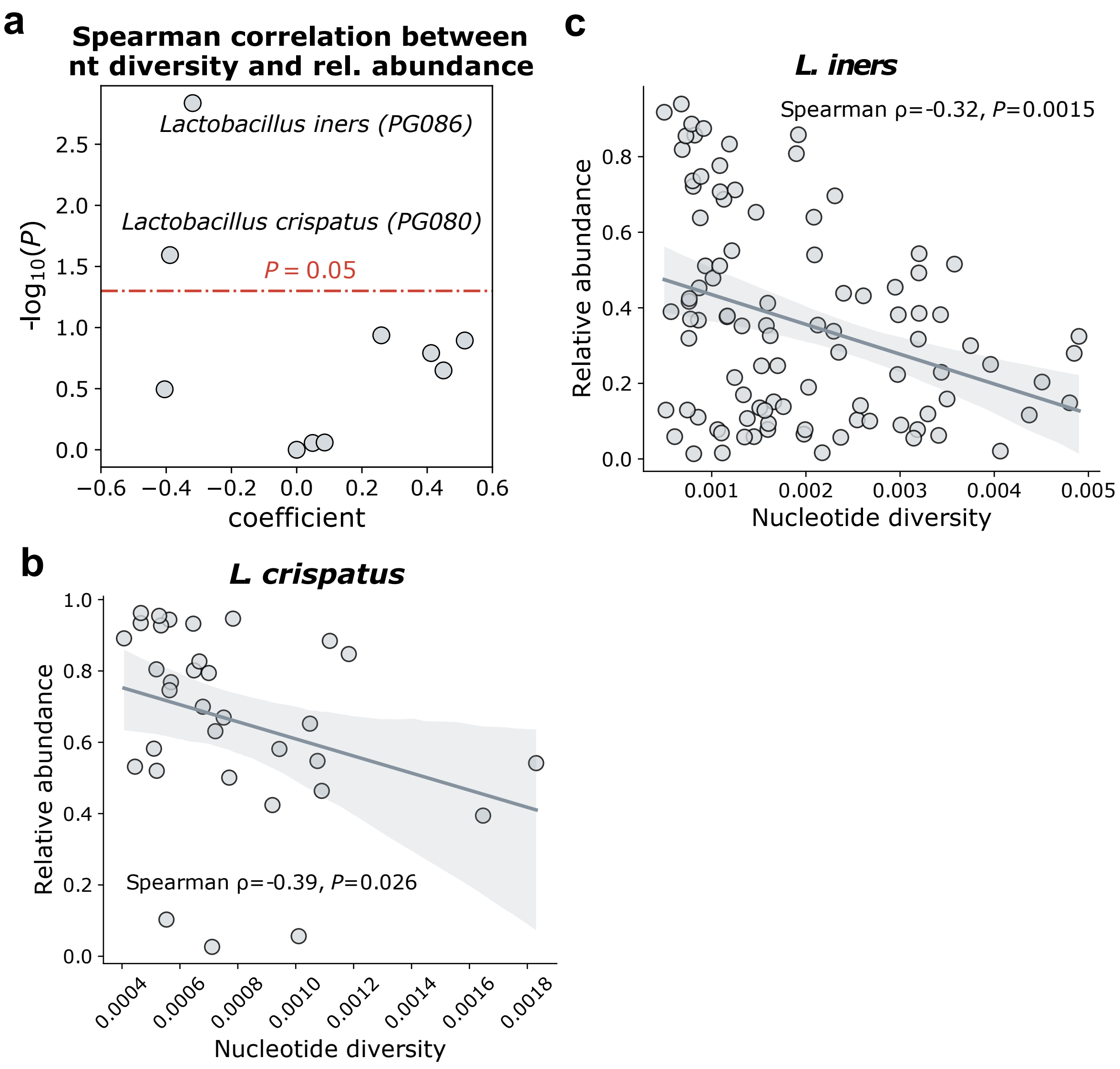


**Supplementary Fig. 6 | Spearman correlation between genome-wide nucleotide diversity and relative abundance of non-*Gardnerella* phylogroups. a.** Volcano plot illustrating the Spearman correlation (significance, y-axis; coefficient, x-axis) between median genome-wide nucleotide diversity and relative abundance along pregnancies. Phylogroups above the red dashed line have a $P < 0.05$. **b,c,** Spearman correlation between median genome-wide nucleotide diversity and relative abundance of *L. crispatus* (b) and *L. iners* (c) along pregnancy. The line and the shaded area depict the best-fit trendline and the 95% confidence interval (mean ± 1.96 s.e.m.) of the linear regression.

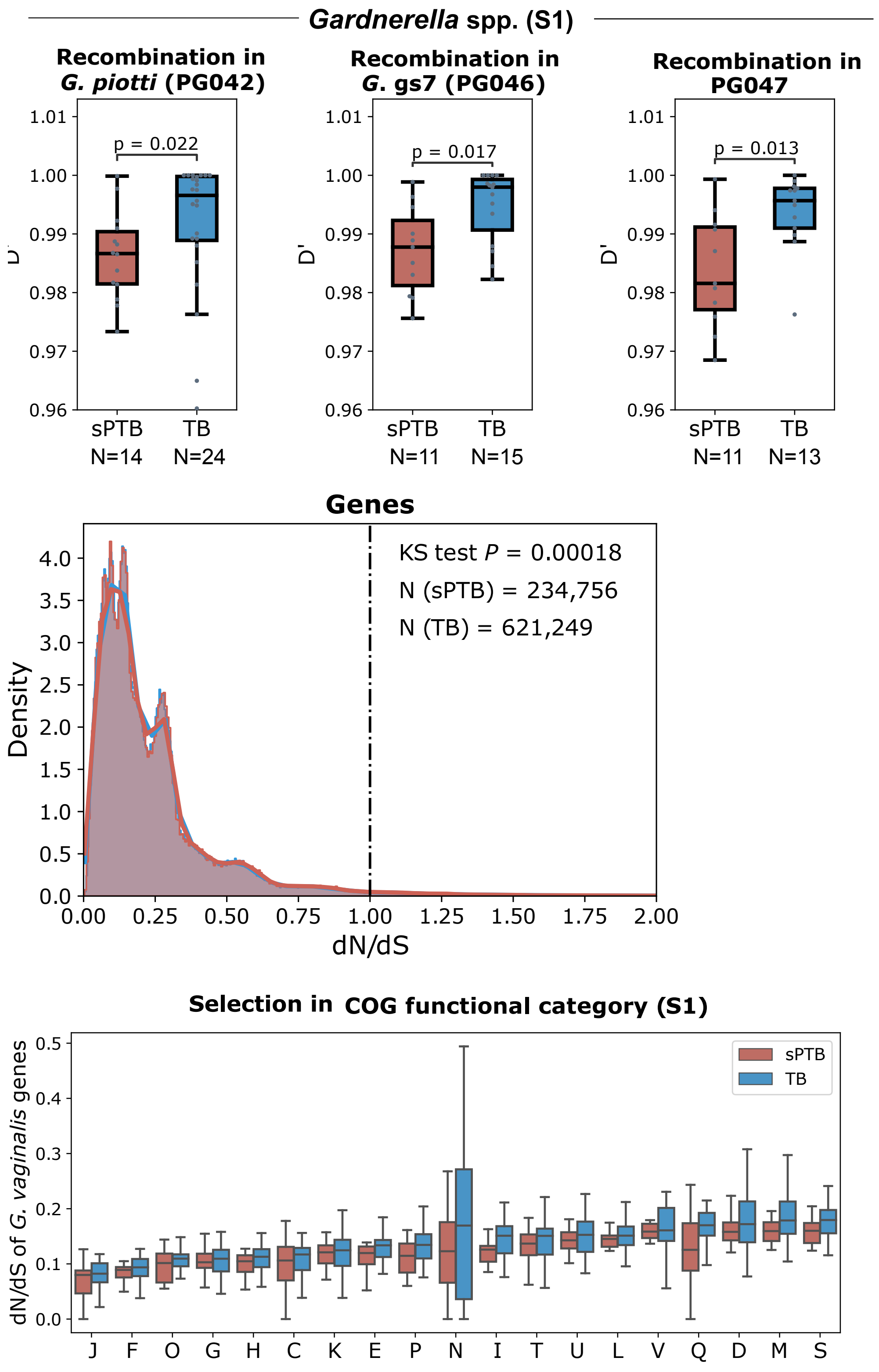


**Supplementary Fig. 7 | Evolutionary forces on the vaginal microbiome. a.** Median D' along the first half of pregnancy (S1) of *Gardnerella* spp. phylogroups compared between sPTB and TB. Lower D' indicates more frequent recombination. **b.** Density of median (along pregnancy) of dN/dS of genes in sPTB (red) and in TB (blue). *P*: two-sided Kolmogorov–Smirnov (KS) test. **c.** dN/dS of *Gardnerella* spp. genes compared between sPTB

and TB by COG functional categories, displayed for the first half of pregnancy (S1). N = 40 and 135 for sPTB and TB, respectively, included in this panel. C, Energy production and conversion; D, Cell cycle control, cell division, chromosome partitioning; E, Amino acid transport and metabolism; F, Nucleotide transport and metabolism; G, Carbohydrate transport and metabolism; H, Coenzyme transport and metabolism; I, Lipid transport and metabolism; J, Translation, ribosomal structure and biogenesis; K, Transcription; L, Replication, recombination and repair; M, Cell wall/membrane/envelope biogenesis; N, Cell Motility, O: Post-translational modification, protein turnover, chaperones; P, Inorganic ion transport and metabolism; Q, Secondary metabolites biosynthesis, transport and catabolism; T, Signal transduction mechanisms; U, Intracellular trafficking, secretion, and vesicular transport; V, Defense mechanisms. Box, IQR; line, median; whiskers, 1.5*IQR; *P*, two-sided Mann-Whitney.

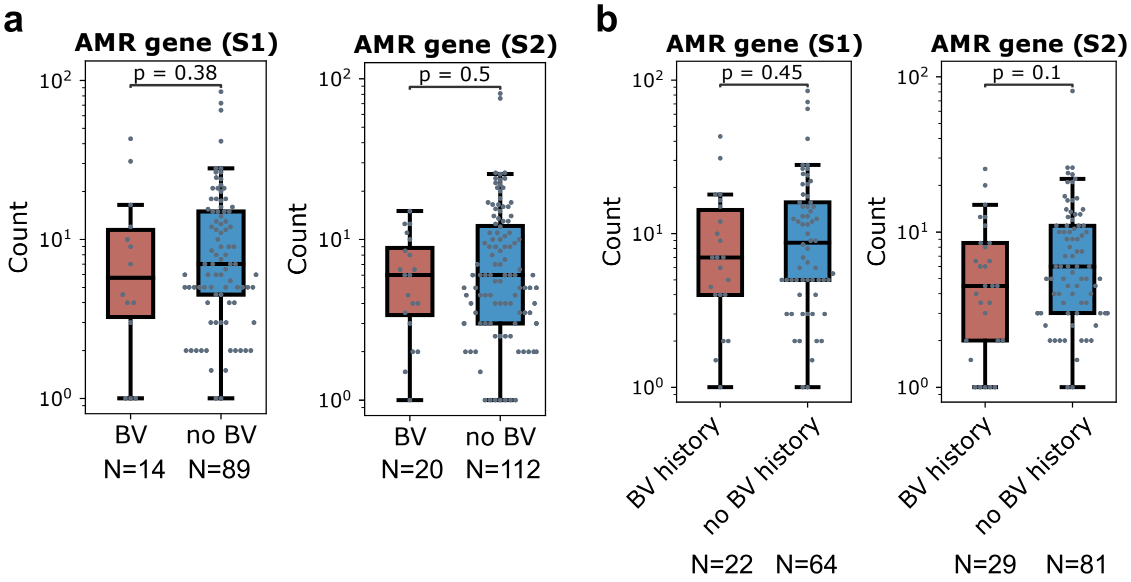


**Supplementary Fig. 8 | Antimicrobial resistance (AMR) gene profiles of the vaginal microbiome are not associated with bacterial vaginosis (BV). a,b,** Median count (along period) of AMR genes in the first (S1) and second (S2) halves of pregnancy, compared between women with and without BV (**a**) and between women with and without BV history (**b**)**.** Box, IQR; line, median; whiskers, 1.5*IQR; *p*, two-sided Mann-Whitney U.

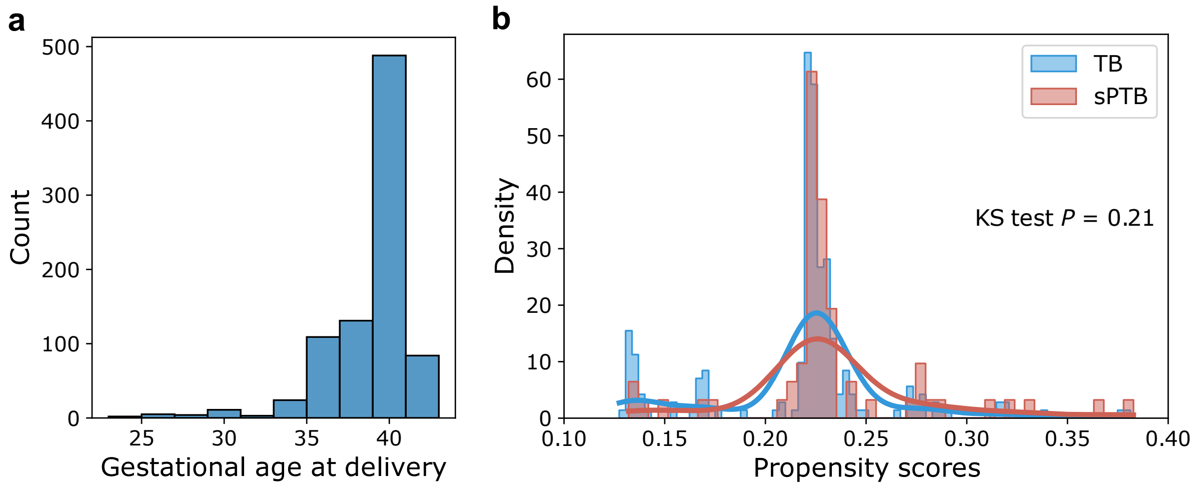


**Supplementary Fig. 9 | Sample summary. a.** Histogram showing the distribution of gestational age of women at delivery. **b.** Distribution of propensity scores of women groups based on income, age, and race using a logistic regression model. The histogram is smoothed using a kernel. sPTB: red, TB: blue, *P*: two-sided Kolmogorov–Smirnov (KS) test.

## Supplementary tables

**Supplementary Table 1** Summary of longitudinal samples collected during pregnancy for women who delivered spontaneous preterm (sPTB) and at term (TB).

| | sPTB | TB | Two-sided Mann-Whitney U *P* |
|---|---|---|---|
| N of samples / woman (mean ± s.d.) | 3.36 ± 1.51 | 3.21 ± 1.13 | 0.83 |
| N of samples / woman for the first half of pregnancy (mean ± s.d.) | 1.68 ± 0.69 | 1.51 ± 0.56 | 0.30 |
| N of samples / woman for the second half of pregnancy (mean ± s.d.) | 2.34 ± 1.10 | 2.14 ± 0.90 | 0.27 |
| Gestational age at the first sample (mean ± s.d.) | 17.38 ± 8.08 | 16.09 ± 7.50 | 0.45 |
| Gestational age at the last sample (mean ± s.d.) | 31.23 ± 5.52 | 32.31 ± 3.66 | 0.72 |
