# Supplementary material for: Microdiversity of the vaginal microbiome is associated with preterm birth
Source: Nat Commun. 2023 Aug 17;14:4997. doi: 10.1038/s41467-023-40719-7 (PMC10435516; doi:10.1038/s41467-023-40719-7)
Supplement: Supplementary file 3 — Description of Additional Supplementary Files [file 41467_2023_40719_MOESM3_ESM.docx]

**Description of Additional Supplementary Files**

**File Name: Supplementary Data 1
Description:** Genome assembly features of representative MAGs for phylogroups and taxonomy.

**File Name: Supplementary Data 2
Description:** eggNOG functional annotation of genes.

**File Name: Supplementary Data 3
Description:** STORMS checklist.
