# Supplementary material for: Microdiversity of the vaginal microbiome is associated with preterm birth
Source: Nat Commun. 2023 Aug 17;14:4997. doi: 10.1038/s41467-023-40719-7 (PMC10435516; doi:10.1038/s41467-023-40719-7)
Supplement: Supplementary file 5 — Reporting Summary [file 41467_2023_40719_MOESM5_ESM.pdf]

nature portfolio

Corresponding author(s): Jingqiu Liao, Tal Korem

Last updated by author(s): Jul 15, 2023

Reporting Summary

Nature Portfolio wishes to improve the reproducibility of the work that we publish. This form provides structure for consistency and transparency in reporting. For further information on Nature Portfolio policies, see our Editorial Policies and the Editorial Policy Checklist.

Statistics

For all statistical analyses, confirm that the following items are present in the figure legend, table legend, main text, or Methods section.

n/a	Confirmed	
☐	☒	The exact sample size (*n*) for each experimental group/condition, given as a discrete number and unit of measurement
☐	☒	A statement on whether measurements were taken from distinct samples or whether the same sample was measured repeatedly
☐	☒	The statistical test(s) used AND whether they are one- or two-sided<br>*Only common tests should be described solely by name; describe more complex techniques in the Methods section.*
☐	☒	A description of all covariates tested
☐	☒	A description of any assumptions or corrections, such as tests of normality and adjustment for multiple comparisons
☐	☒	A full description of the statistical parameters including central tendency (e.g. means) or other basic estimates (e.g. regression coefficient) AND variation (e.g. standard deviation) or associated estimates of uncertainty (e.g. confidence intervals)
☐	☒	For null hypothesis testing, the test statistic (e.g. *F*, *t*, *r*) with confidence intervals, effect sizes, degrees of freedom and *P* value noted<br>*Give P values as exact values whenever suitable.*
☒	☐	For Bayesian analysis, information on the choice of priors and Markov chain Monte Carlo settings
☒	☐	For hierarchical and complex designs, identification of the appropriate level for tests and full reporting of outcomes
☐	☒	Estimates of effect sizes (e.g. Cohen's *d*, Pearson's *r*), indicating how they were calculated

Our web collection on statistics for biologists contains articles on many of the points above.

Software and code

Policy information about availability of computer code

Data collection	No data was collected in this study.
Data analysis	ATLAS pipeline v.2.4.4<br>Trimmomatic v 0.39<br>Bowtie2 v 2.3.5.1<br>metaSPAdes v 3.15.2<br>MetaBAT2 v 2.14.0<br>CheckM v 1.0.9<br>dRep v 3.2.0<br>Prodigal v 2.6.3<br>EggNOG v 5.0<br>GTDB-Tk v 1.5.1<br>RAxML v 8.2.12<br>iTol v 6.3<br>InStrain v. 1.0.0<br>pyani v. 0.2<br>gRodon v. 1<br>PathoFact v 1.0<br>CTF v.1

vegan v. 2.6-4
R v. 3.6.0
Code to replicate all analyses is available from https://github.com/korem-lab/MOMs-PI_microdiversity_2023

For manuscripts utilizing custom algorithms or software that are central to the research but not yet described in published literature, software must be made available to editors and reviewers. We strongly encourage code deposition in a community repository (e.g. GitHub). See the Nature Portfolio guidelines for submitting code & software for further information.

Data

Policy information about availability of data

All manuscripts must include a data availability statement. This statement should provide the following information, where applicable:

- Accession codes, unique identifiers, or web links for publicly available datasets
- A description of any restrictions on data availability
- For clinical datasets or third party data, please ensure that the statement adheres to our policy

The raw sequencing data and metadata were obtained from ref. 1. Data are available under restricted access for ethical and privacy concerns from dbGAP under accession phs001523 [https://www.ncbi.nlm.nih.gov/projects/gap/cgi-bin/study.cgi?study_id=phs001523.v1.p1]. Access can be obtained by a data access request to dbGaP [https://dbgap.ncbi.nlm.nih.gov/aa/wga.cgi], under the purview of the data access committee of the Eunice Kennedy Shriver National Institute of Child Health and Human Development [HD-DAC@mail.nih.gov]. All processed features generated in this study are available95 at Zenodo, doi: https://doi.org/10.5281/zenodo.8150902. The validation dataset32 is available in SRA, under accession PRJNA288562 [https://www.ncbi.nlm.nih.gov/bioproject/?term=PRJNA288562].

Research involving human participants, their data, or biological material

Policy information about studies with human participants or human data. See also policy information about sex, gender (identity/presentation), and sexual orientation and race, ethnicity and racism.

Reporting on sex and gender	All individuals in the study were female. Information regarding gender is not available to us.
Reporting on race, ethnicity, or other socially relevant groupings	Race, ethnicities, and other socially relevant grouping were not used in our analysis.
Population characteristics	Population characteristics have been described in Fettweis et al., Nature Medicine 2019. The cohort is 100% female. Age was reported as 26 (5.68) mean(sd) and 25.9 (5.43) for PTB and TB groups. The race/ethnicity reported was 77.8% African, 13.3% European, 6.7% Hispanic, and 2.2% Native American for the PTB group, and 78.9% African, 14.4% European, 5.6% Hispanic and 1.1% Native American for the TB group.
Recruitment	No recruitment was involved in this study.
Ethics oversight	This is a secondary analysis which was approved by the IRB of Columbia University (AAAS5367).

Note that full information on the approval of the study protocol must also be provided in the manuscript.

Field-specific reporting

Please select the one below that is the best fit for your research. If you are not sure, read the appropriate sections before making your selection.

[x] Life sciences [ ] Behavioural & social sciences [ ] Ecological, evolutionary & environmental sciences

For a reference copy of the document with all sections, see nature.com/documents/nr-reporting-summary-flat.pdf

Life sciences study design

All studies must disclose on these points even when the disclosure is negative.

Sample size	We used all available samples, sample size was determined by the authors of the published study.
Data exclusions	No data was excluded from the analysis.
Replication	There are no experimental findings in this study. We have replicated our main findings in an additional dataset (Fig. 2h, i).
Randomization	This was a secondary analysis of a publicly available dataset, the original study was observational, and there was no intervention. Hence, randomization is not relevant.
Blinding	This was a secondary analysis of a publicly available dataset, the original study was observational, and there was no intervention. Hence, blinding is not relevant.

Reporting for specific materials, systems and methods

We require information from authors about some types of materials, experimental systems and methods used in many studies. Here, indicate whether each material, system or method listed is relevant to your study. If you are not sure if a list item applies to your research, read the appropriate section before selecting a response.

Materials & experimental systems

n/a	Involved in the study
☒	☐ Antibodies
☒	☐ Eukaryotic cell lines
☒	☐ Palaeontology and archaeology
☒	☐ Animals and other organisms
☒	☐ Clinical data
☒	☐ Dual use research of concern
☒	☐ Plants

Methods

n/a	Involved in the study
☒	☐ ChIP-seq
☒	☐ Flow cytometry
☒	☐ MRI-based neuroimaging
